# Effects of live and heat-killed *Bifidobacterium lactis* in rats with induced periodontitis

**DOI:** 10.1590/1678-7765-2025-0634

**Published:** 2026-03-09

**Authors:** Tainá da Silva Tricoly, Thais Aguiar Santos, Camila Lopes Ferreira, Itza Amarilis Ribeiro Pinto, Victoria Clara da Silva Lima, Camilla Magnoni Moretto Nunes, Ana Lia Anbinder, Maria Aparecida Neves Jardini

**Affiliations:** 1 Universidade Estadual Paulista Instituto de Ciência e Tecnologia Departamento de Diagnóstico e Cirurgia São José dos Campos SP Brasil Universidade Estadual Paulista (UNESP), Instituto de Ciência e Tecnologia, Departamento de Diagnóstico e Cirurgia, Divisão de Periodontia, São José dos Campos, SP, Brasil.; 2 Universidade Estadual Paulista Instituto de Ciência e Tecnologia Departamento de Biociência e Diagnóstico Oral São José dos Campos SP Brasil Universidade Estadual Paulista (UNESP), Instituto de Ciência e Tecnologia, Departamento de Biociência e Diagnóstico Oral, São José dos Campos, SP, Brasil.; 3 Universidade de Taubaté Departamento de Odontologia Taubaté SP Brasil Universidade de Taubaté (UNITAU), Departamento de Odontologia, Taubaté, SP, Brasil.

**Keywords:** Periodontitis, Probiotics, Bifidobacterium animalis subsp lactis, Postbiotics

## Abstract

**Background:**

Probiotics and postbiotics have emerged as promising adjunctive therapies in managing periodontal disease. *Bifidobacterium animalis* subsp. *lactis* HN019 has shown antimicrobial and immunomodulatory effects in both experimental and clinical settings when administered orally. However, the systemic impact of this strain, independent of local oral effects, remains unclear.

**Objective:**

To evaluate the systemic effects of live and heat-killed B. animalis subsp. lactis HN019 in a rat model of ligature-induced periodontitis, excluding direct contact with the oral cavity.

**Methodology:**

A total of 32 Wistar rats were randomly assigned to four groups (n=8): control (C), periodontitis only (EP), periodontitis + probiotic (PRO), and periodontitis + postbiotic (POS). Periodontitis was induced by placing a cotton ligature around the cervical region of the lower right first molar, inserted into the gingival sulcus. Treatments were administered via oral gavage for 30 days before and 15 days after periodontitis induction. Alveolar bone loss and periodontal parameters were assessed using micro-computed tomography (microCT) and histomorphometric analysis.

**Results:**

MicroCT revealed that ligature effectively induced periodontitis, reducing BV/TV and Tb.N and increasing Tb.Sp and Po.Tot. Probiotic and postbiotic treatments did not improve outcomes. Bone loss was lowest in the control group, with no differences between EP, PRO, and POS.

**Conclusion:**

Systemic administration of *Bifidobacterium animalis* subsp. *lactis* HN019 or its derived postbiotic resulted in no significant improvements in periodontal outcomes in this experimental model. Further investigations using integrative approaches are needed to better characterize the systemic effects of probiotics and postbiotics.

## Graphical abstract

**Figure:**
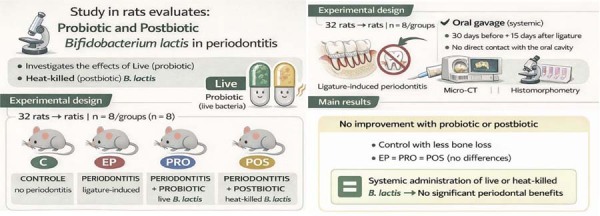

## Introduction

Periodontitis is a chronic, multifactorial disease caused by an imbalance between the host and the oral microbiota, constituting one of the most common oral health problems worldwide.^1,2^ Local consequences include the loss of soft and hard tissues that support the teeth, ultimately leading to tooth loss. Additionally, periodontal diseases may have systemic repercussions, contributing to the progression of conditions such as cardiovascular disease, preterm birth, respiratory disorders, and diabetes-related complications.^3^

Conventional periodontitis treatment involves scaling and root planing (SRP), which aims to mechanically remove biofilm and calculus from the tooth surface. While SRP is considered the gold standard, it is not always effective for all patients, particularly those with systemic conditions or altered immune responses.^4^ Moreover, this approach focuses exclusively on eliminating local etiological factors and does not address the dysregulated host immune-inflammatory response that plays a central role in disease pathogenesis.^5^ In light of this, increasing attention has been directed toward host modulation therapies as adjuncts to periodontal treatment. Several studies have evaluated the use of synthetic and biological agents—including disease-modifying antirheumatic drugs, monoclonal antibodies, probiotics, and nutritional supplements—for their potential to modulate host response.^5-7^

Probiotics are live microorganisms which, when administered in adequate amounts, confer health benefits to the host.^8^ Indirectly, they help modulate local immune responses and non-immunologic defense pathways, regulate mucosal permeability, support systemic immune function, and promote oral colonization by less pathogenic microbial species. Directly, probiotics act on biofilm formation by disrupting bacteria-to-bacteria interactions, compete with oral microorganisms for available substrates, produce antimicrobial compounds that inhibit oral pathogens, and interfere with the binding of microorganisms to proteins involved in biofilm formation.^9^ These multifaceted actions contribute to their potential as adjunctive agents in preventing and managing periodontal diseases due to their antimicrobial and immunomodulatory capacities.^10-12^ The functions of probiotics are highly dependent on the species, strain, and experimental design employed. As a result, drawing robust and generalizable conclusions remains challenging in some systematic reviews.^4,10-12^ This variability helps explain why, despite promising outcomes, probiotics are not yet formally recommended as a treatment option for periodontal disease.^4^

*Bifidobacterium animalis* subsp. *lactis* HN019 is a gram positive, anaerobic, non-spore forming rod shaped bacterium, originally isolated from a yogurt source. It supports gut health by strengthening the intestinal barrier, modulating immune responses and inhibiting pathogen colonization. It also improves gut motility by reducing transit time and increasing bowel movement frequency.^13^ It has also shown promising results as an adjunctive therapy in periodontal disease management. Preclinical studies using oral probiotic administration revealed reduced alveolar bone loss, inflammatory infiltrates, and attachment loss in ligature-induced periodontitis models.^14-16^ These effects were attributed to both antimicrobial activity against key periodontopathogens and immunomodulatory properties, including increased expression of anti-inflammatory cytokines and β-defensins.^8,16^ In human clinical trials, *B. lactis* HN019 was also administered orally, typically via lozenges, as an adjunct to non-surgical periodontal therapy. These studies reported improvements in clinical parameters such as plaque index, bleeding on probing, probing depth, and clinical attachment levels compared with placebo.^17,19^

Although probiotics use is generally considered safe, it is not without risks, particularly in immunocompromised individuals. As an alternative, postbiotics—defined as inactivated microbial cells, cell fractions, or soluble products and metabolites derived from probiotics—have been proposed as therapeutic agents for various diseases, including periodontitis. Their intrinsic stability, lower risk of infection, and ease of storage and handling make them attractive candidates for adjunctive periodontal therapy.^19^ In periodontology, postbiotics have shown antimicrobial and immunomodulatory properties in preclinical models, contributing to reduce inflammation and alveolar bone loss. Although initial clinical findings are encouraging, further standardized trials are needed to confirm their efficacy and establish their role in periodontal therapy.^20^

Probiotic administration route may directly influence its mechanism of action. Oral administration via tablets, lozenges, food, or solutions, allows the microorganisms to come into direct contact with the oral cavity, enabling local effects such as microbial antagonism and modulation of the gingival immune response.^21^ In contrast, gastric gavage delivers the microorganisms directly to the gastrointestinal tract without contact with periodontal tissues; thus, any observed effects are likely mediated by systemic host modulation. Accordingly, this study evaluated the systemic effects of live and heat-killed *B. animalis* subsp. *lactis* HN019 in a rat model of ligature-induced periodontitis, excluding direct contact with the oral cavity.

## Methodology

This study was approved by the Institutional Animal Care and Use Committee (Protocol No. 06-2022) and followed the ARRIVE 2.0 guidelines.^22^ Sample size calculation was based on Oliveira, et al.^15^ (2017) targeting 80% statistical power and a 95% confidence interval to detect significant differences in bone volume (BV) or bone volume fraction (BV/TV) in the furcation area. Accordingly, 32 male Wistar rats (*Rattus norvegicus albinus*), aged three months and weighing 200–350 g, were housed under a 12 h light/dark cycle at 22–24 °C with food and water *ad libitum*. After a 14-day acclimatization period, the animals were randomly allocated into four groups (n=8) according to periodontitis induction and treatment:

- Control group (C): No periodontitis, no treatment
- Experimental Periodontitis group (EP): Periodontitis, no treatment
- Probiotic group (PRO): Periodontitis, treated with viable *B. lactis*
- Postbiotic group (POS): Periodontitis, treated with heat-killed *B. lactis*

Groups C and EP received 300 μL of MRS broth. PRO received 1.2 × 10⁹ CFU/300 μL of viable *Bifidobacterium animalis* subsp. *lactis* HN019 (Aché, Brazil), and POS received the same concentration of heat-inactivated bacteria. All treatments were administered via gavage to avoid probiotic contact with the oral cavity.

### Probiotic and postbiotic preparation

*B. lactis* HN019 was cultured on De Man, Rogosa and Sharpe (MRS) agar at 37 °C. Bacterial suspension was standardized by spectrophotometry at 625 nm.1^3^ For postbiotic preparation, the final bacterial suspension was autoclaved at 121 °C for 15 minutes.^23^ Inactivation was confirmed by plating the suspension on MRS agar and incubating for 3 days to verify absence of colony growth.

### Experimental design

Treatment was administered for 30 consecutive days prior to periodontitis induction and continued for 15 days after, when euthanasia occurred. On day 30, animals in the EP, PRO and POS groups were anesthetized via intramuscular injection of ketamine (100 mg/kg, Dopalen) and xylazine (10 mg/kg, Anasedan). Periodontitis was induced by placing a cotton ligature around the cervical region of the lower right first molar, gently inserted into the gingival sulcus.^22^ Control animals were anesthetized and manipulated without ligature placement to simulate surgical stress. Analgesia was provided with oral dipyrone (25 mg/kg, once daily) for three days post-procedure.

On day 45, all animals were euthanized by triple-dose anesthesia. Right hemimandibles were collected and fixed in 10% neutral-buffered formalin for 48 h and then transferred to 10% formic acid for decalcification. Left hemimandibles were stored in 70% ethanol for microcomputed tomography (microCT) analysis.

### Microcomputed tomography

All samples were scanned using a high-resolution micro-computed tomography system (SkyScan 1172, Bruker microCT, Kontich, Belgium) at 59 kV and 165 µA, with a 0.5 mm aluminum filter and an isotropic voxel size of 7.95 µm. Scanning was performed with a rotation step of 0.6° and exposure time of 605 ms per projection. Images were reconstructed on NRecon (version 1.6.6.0) and aligned in transverse, longitudinal, and sagittal planes using DataViewer (version 1.4.4). The region of interest (ROI) was defined as the interradicular area between the mesial, distal, buccal, and lingual aspects of the tooth. Coronal limit was set at the furcation, and the apical limit extended 135 tomograms toward the root apex. ROI selection was performed every 10 tomograms throughout the volume using CTAn software (version 1.12.4.0). Parameters measured included bone volume fraction (BV/TV), trabecular number (Tb.N), trabecular separation (Tb.Sp) and total porosity (PO.Tot).

### Histomorphometric analysis

Paraffin-embedded sections (5 μm) of the collected hemimandibles were stained with hematoxylin and eosin (H&E) and scanned at 50x magnification. Furcation bone loss was quantified by measuring the area corresponding to the periodontal ligament space between the mesial and distal roots in 10 semi-serial sections per specimen. Interproximal connective tissue attachment loss was assessed by measuring the distance from the cementoenamel junction (CEJ) to the apical end of the junctional epithelium. Linear bone loss was measured from the CEJ to the alveolar bone crest at the distal aspect of the first molar. All histomorphometric analyses used ImageJ software (ImageJ 1.31p. National Institutes of Health, Bethesda, MD, USA).

### Statistical analysis

Data normality was assessed using the Kolmogorov-Smirnov test. Parametric data analysis employed one-way ANOVA followed by Tukey’s post hoc test. Non-parametric data were analyzed using the Kruskal-Wallis test followed by Dunn’s multiple comparisons test. A significance level of *p*<0.05 was adopted. All statistical analyses were performed on GraphPad Prism version 8.0 (GraphPad Software, San Diego, CA, USA).

## Results

MicroCT analysis evaluated five parameters: BV/TV, Tb.N, Tb.Sp and Po.Tot. Ligature placement effectively induced periodontitis, as evinced by the significant reduction in BV/TV and Tb.N, and the increase in Tb.Sp, Po.Tot, alveolar bone loss (ABL), and attachment loss in the periodontitis groups compared with control.

BV/TV and Tb.N yielded higher values in control when compared with the other groups. Inversely, Tb.Sp and Po. Tot presented lower values in control when compared with the other groups.

**Figure 1:**
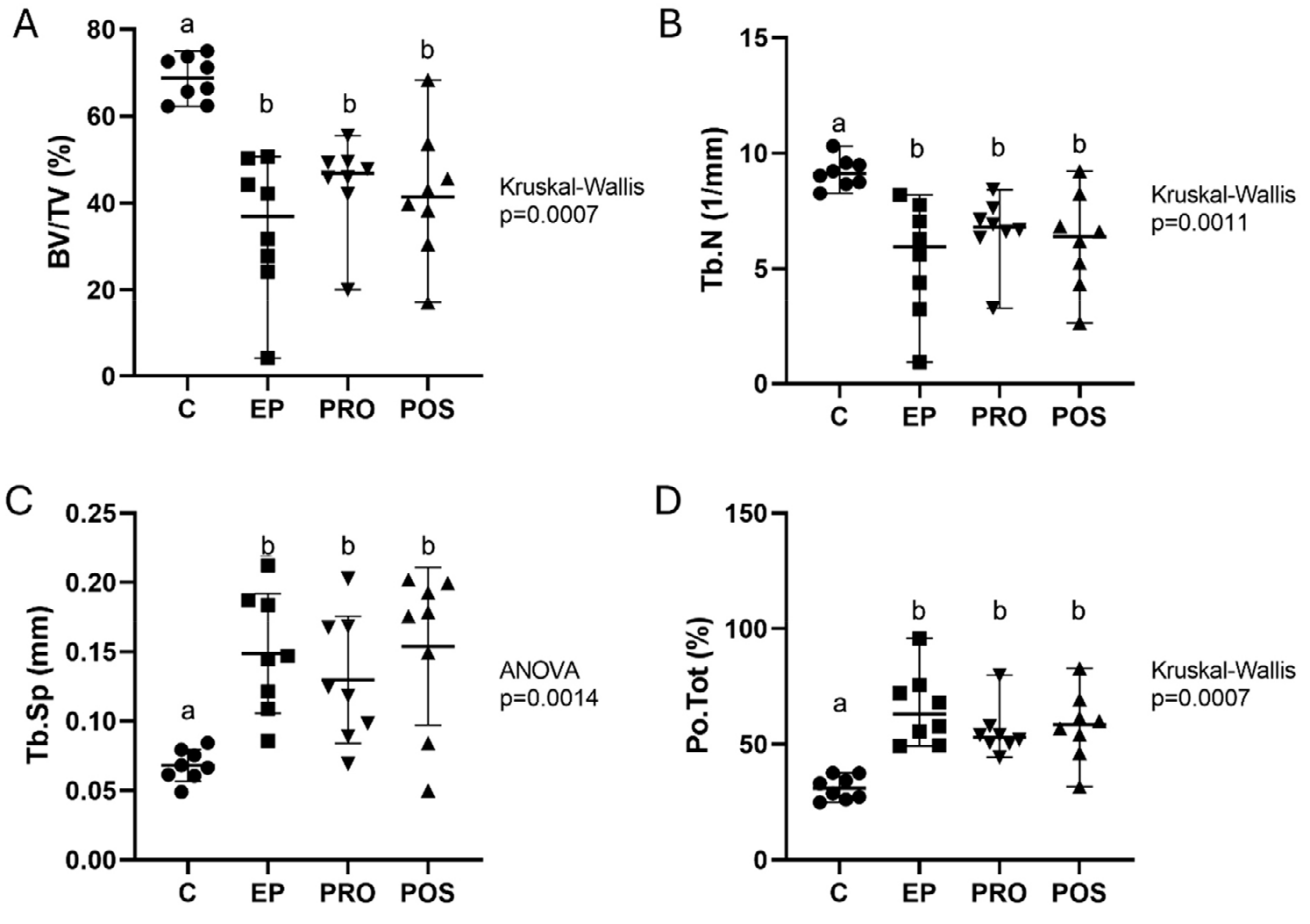
Micro-CT analysis of alveolar bone microarchitecture in the furcation area. Dot plots showing bone volume fraction (BV/TV, a), trabecular number (Tb.N, b), trabecular separation (Tb.Sp, c), and total porosity (Po.Tot, d). Each dot represents one experimental unit. Data are presented as median and range in a, b, and d, and as mean and standard deviation in c. Different uppercase letters over the groups indicate statistically significant differences between groups (p<0.05); in all parameters, the control group (C) differed from the EP, PRO, and POS groups, which did not differ from each other. n=8 per group (ANOVA followed by Tukey’s test; Kruskal–Wallis followed by Dunn’s test).

However, neither probiotic nor postbiotic treatment was effective in improving these periodontal disease parameters (Figure 1).

**Figure 2:**
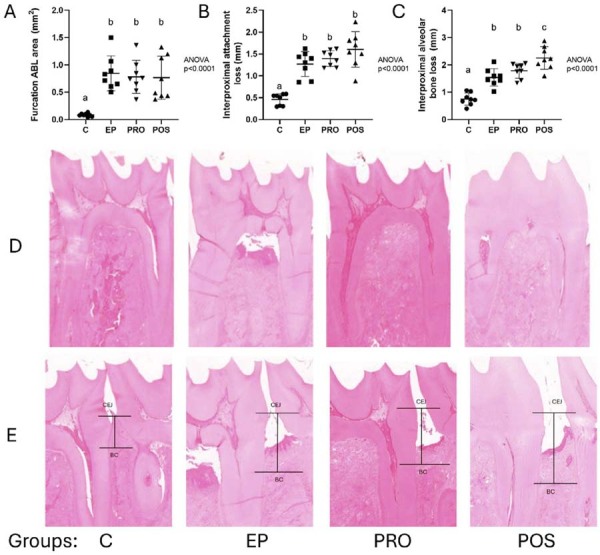
Histomorphometric analysis of periodontal breakdown. Dot plots showing furcation alveolar bone loss (a), interproximal attachment loss (b), and interproximal alveolar bone loss (c). Each dot represents one experimental unit. Data are presented as mean and standard deviation. Different uppercase letters over the groups indicate statistically significant differences between groups (p<0.05). Statistical analysis was performed using ANOVA followed by Tukey’s test. n=8 per group. Representative H&E-stained sections (d) illustrating the interradicular and interproximal regions (e) of the mandibular first molars in the different experimental groups. CEJ, cementum–enamel junction; BC, bone crest.

Histomorphometric analysis of bone loss in the furcation area showed significantly greater bone loss in the EP, PRO, and POS groups compared with control, without differences between the experimental groups (Figure 2).

## Discussion

Periodontitis is a multifactorial disease driven by a dysregulated interaction between the oral microbiota and the host immune system. Conventional treatment strategies typically target either microbial control—via SRP to remove biofilm and calculus—or modulation of the host response.^2,4^ In this context, probiotics represent a promising therapeutic adjunct, as they exert beneficial effects on both fronts. They can inhibit pathogenic bacteria growth and disrupt biofilm formation while modulating immune responses both locally and systemically. Locally, probiotics may suppress inflammation by influencing dendritic cell activation and toll-like receptor signaling, reducing production of pro-inflammatory cytokines.^6,10,13^ Systemically, they contribute to mucosal integrity maintenance by enhancing tight junction protein expression and reducing gut permeability, thereby preventing translocation of microbial components. Additionally, their impact on insulin and lipid metabolism further supports their role in mitigating systemic inflammation.^20^

When administered orally—whether through lozenges, drinking water, or food—probiotics first come into contact with the oral cavity. This route enables both local effects (such as antimicrobial and immunomodulatory actions) and systemic effects on periodontal tissues, as the probiotics are subsequently swallowed. In experimental models, oral administration is commonly performed via drinking water or feed, which may prolong probiotic exposure to the oral cavity but does not allow precise control over the ingested dose.^14-16^ In contrast, gavage ensures that all animals receive an identical amount of probiotic and that its action is exclusively systemic, since it bypasses contact with the oral environment. We chose this approach in the present study to evaluate systemic effects only. Given the favorable outcomes reported with postbiotics in periodontitis,^20^ research also shows that microbial viability is not always required for beneficial effects. Thus, this study was designed to assess whether the observed outcomes are due to systemic actions of the probiotic/posbiotic intervention.

We selected *Bifidobacterium lactis* HN019 due to its well-documented periodontal benefits, as reported in several experimental studies^14-16^and clinical trials^15,16,23^when administered orally. We adopted the same experimental timeline previously established by Messora, et al.^14^ (2013) and Moraes, et al.^23^ (2020), consisting of probiotic administration starting 30 days before periodontitis induction and continuing for approximately 14–15 days after it. This protocol was originally designed to evaluate the preventive and early therapeutic effects of probiotics in ligature-induced periodontitis. An important methodological difference between those studies and the present investigation is the administration route. While Messora, et al.^14^ (2013) and Moraes, et al.^23^ (2020) administered the probiotic via oral intake, we delivered the probiotic and postbiotic by gavage.

Systemic probiotic administration did not yield the expected improvements in periodontal parameters, suggesting that local antimicrobial and immunomodulatory effects may be more relevant than systemic ones. Supporting this observation, Araújo, et al.^24^ (2023) administered the same strain in a model of experimentally induced periapical disease for up to 42 days and found no significant differences between treated and untreated groups. Conversely, other probiotic species and strains have shown beneficial systemic effects in apical periodontitis models.^25^

Clinical efficacy of probiotics is not solely determined by their strain-specific properties, but also critically depends on the dosage and viability of the microorganisms at time of consumption. Evidence suggests that to exert health benefits, probiotics must be administered in sufficient amounts to survive the gastrointestinal transit and reach the target sites in effective concentrations.^11,12,21^ This requirement is underscored by the observation that beneficial effects—such as immunomodulation, antimicrobial activity, and barrier function enhancement—are often dose-dependent. Higher doses, for example, are more likely to result in faecal recovery of viable strains and elicit measurable physiological responses such as increased production of anti-inflammatory cytokines or modulation of immune cell activity.^21^ Nonetheless, postbiotics derived from certain strains have shown beneficial effects when administered orally for periodontitis^20,23^or systemically for other conditions like osteopenia.^26^ In our study—the first to evaluate a postbiotic derived from *B. lactis*—the absence of systemic effects suggests that the viability of the microorganism is not a determining factor in this context.

One study limitation concerns the lack of analysis of intestinal microbiota. Considering the findings reported by Kayser, et al.^27^ (2024) who observed that both live and heat-treated *Bifidobacterium animalis* subsp. *lactis* CECT 8145 induced significant shifts in the gut microbial composition and functional pathways, changes in the microbiome may have occurred in our model as well. Absence of microbiota profiling restricts our ability to determine whether the lack of systemic effects observed was due to insufficient modulation of gut microbial communities or other host-related factors. Future studies should incorporate microbiome and metabolomic analyses to better elucidate the mechanisms underlying the systemic or local actions of postbiotic interventions.

In summary, systemic administration of *Bifidobacterium animalis* subsp. *lactis* HN019 or its derived postbiotic resulted in no significant improvements in periodontal outcomes in this experimental model. These findings indicate that, under the conditions tested, neither the probiotic nor the postbiotic exerted a protective effect against periodontal breakdown. Although our use of a validated animal model and a standardized dosing protocol strengthens the reliability of the results, the absence of gut microbiota analysis represents an important limitation. Further investigations using integrative approaches are needed to better characterize the systemic effects of probiotics and postbiotics.
